# Early Trends to Show the Efficacy of Cordyceps militaris in Mild to Moderate COVID Inflammation

**DOI:** 10.7759/cureus.43731

**Published:** 2023-08-18

**Authors:** Siddharth Dubhashi, Sagar Sinha, Sankalp Dwivedi, Jaishree Ghanekar, Sameer Kadam, Parineeta Samant, Vibha Datta, Sarman Singh, Irshad H Chaudry, Padma Gurmet, Harshawardhan Kelkar, Rakesh Mishra, Sagar Galwankar, Amit Agrawal

**Affiliations:** 1 Surgery, All India Institute of Medical Sciences, Nagpur, Nagpur, IND; 2 Emergency Medicine, Mahatma Gandhi Mission (MGM) Medical College and Hospital, Navi Mumbai, IND; 3 General Surgery, Maharishi Markandeshwar (MM) Institute of Medical Sciences and Research, Mullana, IND; 4 Internal Medicine, Mahatma Gandhi Mission (MGM) Medical College and Hospital, Navi Mumbai, IND; 5 Cardiovascular Surgery, Mahatma Gandhi Mission (MGM) Medical College and Hospital, Navi Mumbai, IND; 6 Biochemistry, Mahatma Gandhi Mission (MGM) Medical College and Hospital, Navi Mumbai, IND; 7 Pathology, All India Institute of Medical Sciences, Nagpur, Nagpur, IND; 8 Medical Science and Engineering Research (MEDSER) Center, Indian Institute of Science Education and Research (IISER), Bhopal, IND; 9 Surgery, University of Alabama, Birmingham, USA; 10 Miscellaneous, National Institute of Sowa-Rigpa, Leh, IND; 11 Medicine, Ayurarogya, Kelkar Hospital, Nashik, IND; 12 Neurosurgery, All India Institute of Medical Sciences, Bhopal, Bhopal, IND; 13 Emergency Medicine, Florida State University College of Medicine, Sarasota, USA

**Keywords:** cytokines, cordyceps militaris, covid-19, cordyceps, plant based immunotherapy

## Abstract

Background/objective

Cordyceps enhances animal survival against influenza by boosting the immune system. In animal studies, it also had anti-inflammatory and preventive properties. Cordyceps stimulates the immune system by increasing the activity and production of various immune cells. Some studies have shown the role of Cordyceps in the novel SARS-CoV-2 virus responsible for the COVID-19 pandemic, in addition to other respiratory diseases caused by the Picorna viruses, SARS-CoV, MERS-CoV, and Influenza viruses. However, it remains unknown whether this food supplement is safe and has anti-inflammatory effects in patients with COVID-19. Therefore, the objectives of this study were to evaluate the use and efficacy of Cordyceps capsules as an adjunct to standard treatment in patients with mild (symptomatic) to moderate COVID-19 infection.

Methods

A randomised, double-blind, placebo-controlled study was conducted to evaluate the efficacy and safety of Cordyceps capsules (a food supplement) 500 mg as adjuvant therapy in patients with COVID-19. The rationale for dose selection was as per the existing evidence from toxicity studies. The inclusion criteria were patients with either a mild or moderate COVID-19 infection. Clinical features suggestive of dyspnoea or hypoxia, fever, and cough, including SpO_2_ <94% (range 90-94%) on room air and a respiratory rate ≥24 per minute, were also included.

Results

Sixty-five patients were recruited for the study, with 33 in the Cordyceps group and 32 in the placebo group. Out of 58 evaluable patients, 33 recovered on day 5, 49 on day 10, and 58 on days 16 and 30. The recovery of patients steadily increased from 56.9% on day 5 to 100% on day 30. The time to clinical recovery was shorter in the Cordyceps group than in the placebo group (mean 6.6 vs. 7.3 days; p > 0.05) overall and for mild disease. However, there was no difference in the time to recovery (time from day 1 to the resolution of all symptoms) for moderate disease. A lower frequency of normal chest X-rays on day 1 and a higher number on day 16 in the treatment group than in the placebo group suggest an improvement in the number of normal chest X-rays with Cordyceps. Significant changes were seen in biomarkers MCPIP, CxCL10, and IL-1β for overall (both mild and moderate patients) on days 5 and 10 as compared to baseline, and in biomarkers CRP and CxCL10 in moderate category patients on days 5 and 10, respectively. There were no statistically significant changes in IL-6, ferritin, lactate dehydrogenase (LDH), C-reactive protein (CRP), or D-dimer levels between baseline and day 5/10 in patients taking Cordyceps capsules and also between the treatment and placebo groups.

Conclusion

Cordyceps capsules administered at a dose of 500 mg three times a day along with supportive treatment showed effectiveness in patients with mild to moderate COVID-19 infection, as evidenced by the proportionately higher number of recoveries on day 5, the relatively shorter time for improvement of clinical symptoms, and the proportionately higher number of patients showing negative RT-PCR tests on day 10. Thus, Cordyceps appears to be a safe immunological adjuvant for the treatment of patients with mild-to-moderate COVID-19. Future studies with a larger sample size would shed more light on the evidence, as there are limitations in the generalizability of the results from the present study due to the small sample size.

## Introduction

The pathophysiology of COVID infection revolves around cytokine release syndrome (CRS). Inflammatory cytokines and chemokines such as interleukin-6 (IL-6), interleukin-1β (IL-1β), induced protein 10 (IP10), monocyte chemoattractant protein-1 (MCP-1), and tumour necrosis factor (TNF) are significantly elevated in this condition [1-3]. Immunological dysregulation is noted in terms of lymphopenia and decreased regulatory T-cells in critical cases. Severe forms of the condition progress to acute respiratory distress syndrome (ARDS), which can prove fatal. Uncontrolled inflammation can lead to multiorgan failure. Inflammatory cytokine blockade is an important strategy for the control of coronavirus disease (COVID)-related cytokine release syndrome [3-6]. Previous coronavirus infections include severe acute respiratory syndrome (SARS) and Middle East respiratory syndrome (MERS). However, there are significant immunological differences in SARS-CoV-2 despite belonging to the same family of coronaviruses. [7] The immunological differences are responsible for different incubation periods, severity of disease, R naught values, and case fatality ratios among the different coronavirus diseases [8].

Cordyceps is a type of fungus that has long been used in traditional Chinese medicine as tinctures and teas for the treatment of various diseases. There are mainly two species that are used for treatment: *Cordyceps sinensis* and *Cordyceps* militaris. Cordyceps has an effect on the immune system. Cordyceps capsules are marketed as an immunity booster by Ambrosia Food Farm Co. and given as a food supplement (FSSAI registration number: 22619035000547). Each capsule of Cordyceps is 500 mg and contains adenosine 0.96 mg and cordycepin 4.5 mg. It stimulates the immune system through several processes, such as increasing the activity of macrophages, a type of white blood cell; increasing the activity of natural killer cells; stimulating T-cells; increasing the numbers of CD4+ and CD8+ cells; and increasing the production of IFN-γ [9].

The anti-inflammatory potential of Cordycepin has been evident in many published reports. Cordycepin downregulates inducible nitric oxide synthase (iNOS), cyclooxygenase 2 (COX-2), and TNF-α gene expression via the suppression of nuclear factor-κB (NF-κB) activation and phosphorylation of protein kinases, i.e., Akt and p38. [10] Cordycepin down-regulates the gene expression of M1 cytokines (IL-1β, TNF-α) and chemokines (CX3CR1, RANTES) and increases the expression of IL-10, IL-1ra, and TGF-β [11]. Literature evidence indicates the anti-viral activity of Cordyceps against Picorna viruses (RNA viruses), which is mainly due to inhibition of RNA synthesis [12], two of the human coronavirus infections, i.e., SARS-CoV and MERS-CoV [13], and the novel coronavirus SARS-CoV-2, which is responsible for the COVID-19 pandemic [14]. Two mouse studies show that Cordyceps extract and its isolated active components may help fight against the influenza virus, which is responsible for the flu. In both of these studies, Cordyceps increased immune system activity and improved the survival rate in mice [15]. Cordyceps has been shown to help protect against the flu in animal studies, which can help humans get the same health benefits. Several studies suggest that Cordyceps helps protect the lungs, primarily through its anti-inflammatory effects [16]. A study of 120 patients diagnosed with moderate to severe asthma found that those taking Cordyceps supplements reported better lung function and quality of life. The treatment group indicated a significant increase in Asthma Quality of Life Questionnaire (AQLQ) scores and lung function compared with the control group. The expression levels of the inflammation markers IgE, ICAM-1, IL-4, and MMP-9 in the serum were decreased and IgG increased in the treatment group compared with the control group [17]. According to another study of 60 asthma patients, those taking Cordyceps supplements had lower lab markers for airway inflammation compared to those not taking the supplement [11]. In rats, Cordyceps reduced airway thickening, inflammatory cell buildup, and cytokine production [18]. Some animal studies suggest that Cordyceps may help protect the lungs from damage caused by cigarette smoking [19,20]. Some studies suggest the role of traditional medicine in regulating MMP levels [21].

The objectives of the present study were to evaluate if the use of Cordyceps capsules as an adjuvant therapy to standard treatment of SARS-CoV-2 infection is safe, if it has any anti-inflammatory effects, and if it hastens the recovery of patients with mild (symptomatic) to moderate SARS-CoV-2 infection.

## Materials and methods

Methods

This was a randomised, double-blind, placebo-controlled study conducted to evaluate the efficacy and safety of Cordyceps capsules (food supplement) as adjuvant therapy in patients with COVID-19. The place of the study was MGM Hospital Navi Mumbai, and the time period of the study was October 2020 to March 2021. Each capsule of Cordyceps is 500 mg and contains adenosine 0.96 mg and cordycepin 4.5 mg. 500 mg of Cordyceps capsules or placebo were administered three times a day after food (e.g., breakfast, lunch, and dinner) for 15 days. Cordyceps capsules or placebo were administered at approximately the same time each day as an add-on to the standard therapy.

Rationale for Dose Selection

In a toxicity study, once-daily oral administration of freeze-dried *C. militaris* mycelium powder for 90 consecutive days was well tolerated in rats at dose levels of 2000 mg/kg/day, 3000 mg/kg/day, and 4000 mg/kg/day. The NOAEL of *C. militaris* mycelium is 4000 mg per kg BW per day for male and female SD rats. Further, the [rm1] data did not reveal any significant toxicity that would preclude the use of Cordyceps in patients with COVID-19 infection. Previous clinical studies in other conditions have used 1-3 g/day as the dosage for most Cordyceps extracts. Most supplements contain 600-1,000 mg of the extract per capsule. One study gave participants 1.2 g of Cordyceps supplement three times a day for three months with no reported safety concerns [16]. Early results indicate that consuming 1.5 g of Cordyceps powder daily for three months could protect a person from chronic rhinitis and the common cold without any side effects. The 1.5 g dose per day for 15 days was a sufficient dose for the desired effect in patients with COVID-19 infection [17].

The study was approved by the Institute Review Board. The study was conducted following the standards of Good Clinical Practice outlined in the International Conference on Harmonisation of Technical Requirements for Registration of Pharmaceuticals for Human Use (ICH) E6 Tripartite, the Indian Council of Medical Research (ICMR) Ethical Guidelines for Biomedical Research guidelines, the current revision of the Declaration of Helsinki, and applicable regulatory requirements. The study was registered prospectively on the Clinical Trial Registry of India.

Inclusion and exclusion criteria

The patients were included based on the inclusion and exclusion criteria mentioned in Table 1.

**Table 1:** Patient selection criteria *Mild symptomatic COVID-19 infection was defined as patients with uncomplicated upper respiratory tract infection. The patient had mild symptoms such as fever, cough, sore throat, nasal congestion, malaise, and headache, without evidence of breathlessness or hypoxia (normal saturation SpO_2_ ≥ 95%). **Moderately symptomatic COVID-19 infection was defined as pneumonia with no signs of severe disease defined as patients in respiratory distress (RR ≥ 30/min, SpO_2_<90% on room air) or patients requiring oxygen more than 8 L/min at the time of randomization. Clinical features are suggestive of the presence of dyspnoea or hypoxia, fever, and cough, including SpO_2_ <94% (range 90-94%) on room air and a respiratory rate of ≥24 per minute and less than 30.

S. No	Inclusion criteria	Exclusion criteria
1	Patients with either mildly symptomatic* or moderately symptomatic** COVID-19 infection.	Patients who refused to give consent for participation.
2	Male and female patients aged ≥18 years.	Severe category COVID-19 patients were defined as those with respiratory distress (RR ≥ 30/min, SpO_2 _< 90% on room air) or patients requiring oxygen more than 8 L/min at the time of randomisation.
3	Patients with or without co-morbidities (stable diabetes or/and hypertension with medications).	Patients with asymptomatic infections, and patients who were allergic to Cordyceps products.
4	Voluntary willingness to provide written informed consent before participation in the trial.	Patients already enrolled in another intervention trial, patients who receive immunomodulant therapy like Tocilizumab or convalescent plasma or high-dose pulse steroid therapy, autoimmune diseases such as multiple sclerosis (MS), systemic lupus erythematosus (SLE) rheumatoid arthritis (RA) or other conditions.
5	Male patients who were surgically sterile or willing to agree to remain completely abstinent or use barrier contraceptive measures and refrain from donating sperm for 12 weeks.	Patients with any bleeding disorder, e.g., haemophilia and von Willebrand disease, patients in whom the surgeries were planned during the study.
6	Females should have non-childbearing potential, either surgically sterile or postmenopausal. Women should have either a history of no menses for at least 12 months or a documented bilateral oophorectomy with or without hysterectomy.	Pregnant or breastfeeding, a concurrent condition that would have jeopardized compliance with the protocol, inability or unwillingness to comply with study and/or follow-up.
7	Females of childbearing potential agreed to use effective contraceptive measures for a period of 4 weeks and willingness and able to comply with trial and follow-up procedures.	Patients who had severe cardiac involvement secondary to COVID inflammation might present as radiological densities and prior history of pulmonary tuberculosis.

Demographics and medical history were obtained by the investigator during the screening period. After obtaining written informed consent, patients who met all of the inclusion criteria and none of the exclusion criteria were randomised to one of the two groups to receive either Cordyceps capsules as an adjuvant therapy to standard treatment protocol (Arm 1 = 33) or placebo plus standard treatment protocol (Arm 2 = 32) for the treatment of patients with mild (symptomatic) or moderate SARS-CoV-2 infection. All mild patients received the standard of care treatment that included vitamin C, zinc supplements, and antibiotics such as azithromycin or cefixime as per the institutional protocol in both groups. In both groups, patients with moderate disease received antiviral treatment such as injections of remdesivir, anti-inflammatory agents such as prednisolone, and anti-coagulants such as low molecular weight heparin, vitamin C, and trace elements as per the institutional protocol. Individual patients’ participation was for 30 days. For 15 days, either 500 mg of Cordyceps capsules or placebo were taken orally three times per day after meals (such as breakfast, lunch, and supper). Cordyceps capsules or placebo were administered orally at approximately the same time each day.

**Figure 1:**
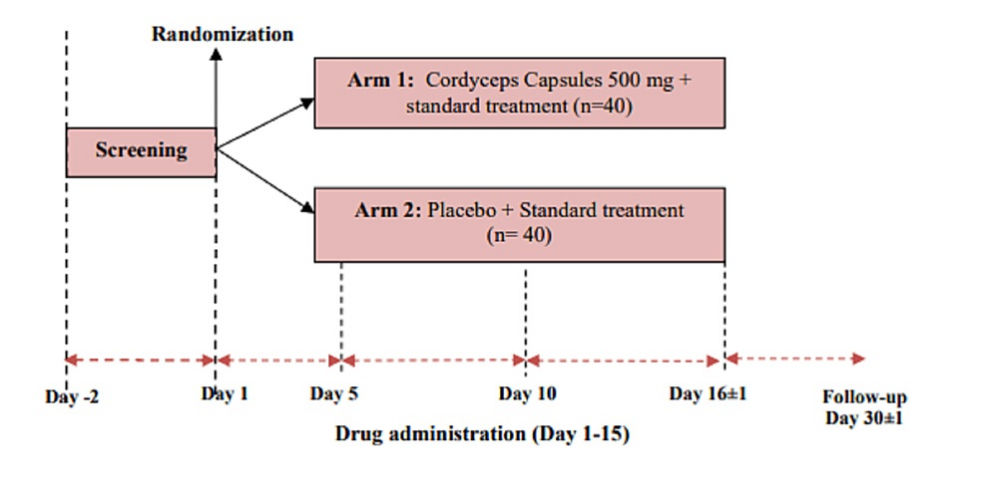
Study flow diagram with number of visits

All visits were done in an ambulatory setting for both mild and moderate categories, unless the patients with moderate infections were required to be hospitalised. For hospitalised patients, if the patient was discharged before day 15, then the patient was followed up on day 16. The 30-day/1-month follow-up was mostly done via telephone. During the hospitalisation period, on all the other days than those mentioned for trial-related investigations, vitals and physical examinations, compliance, and disease evaluation were done as per hospital policy. The enrolled patients were followed for a period of 30 days. The duration of treatment was 15 days. Every effort was made to encourage the patient’s compliance with the dosage regimen as per protocol. All patients were instructed to return their unused drugs to the investigator as applicable. Compliance was assessed based on supplies dispensed, consumed, and returned. A compliance rate of less than 80% was defined as poor compliance. The patients were requested to visit the study centre on their due dates. Apart from this, patients were requested to visit the centre anytime if any adverse event occurred. The visit and study flow diagram is shown in Figure 1.

Evaluation parameters

Evaluation parameters for hastening recovery were as follows. (i) Time to improvement of clinical symptoms is defined as the time from day 1 (baseline) to change of symptoms from moderate to mild/recovery/asymptomatic for moderate patients, and mild to recovery/asymptomatic for mild patients. The time to recovery of clinical symptoms is defined as the time from day 1 (baseline) to the resolution of all symptoms. (ii) The proportion of the patients with a negative COVID-19 test (2019-nCoV RT-PCR) by day 10; (iii) improvement in oxygenation indices by day 10 of treatment in patients with the moderate category; (iv) reduction of inflammatory markers [IL-1, IL-6, MCP-1, IP-10, ferritin, lactate dehydrogenase (LDH), C-reactive protein (CRP), D-dimer, and iNOS] on days 5 and 10 as compared to baseline; (v) the proportion of the patients having IgG antibodies on days 16; (vi) length of hospital stay for hospitalised patients.

Evaluation parameters for safety are (i) haematology and biochemistry tests were performed on screening/baseline, day 5, and day 16; (ii) demographics and medical history: all concurrent medications used before the first dose of the study drug through the follow-up visit were recorded; (iii) the patient’s vitals were recorded at each study visit, and an ECG was recorded at screening/baseline (day 1) and on day 16; (iv) a complete physical examination was done at screening. On subsequent days, an abbreviated examination was carried out. (v) Arterial blood gas (ABG) analysis was carried out in moderate category patients on screening/baseline, day 5, and days 10 and 16; monitoring of adverse events (from screening until the end of treatment assessment); and serious adverse events throughout the study. Events occurring after the treatment were called treatment-emergent adverse events (TEAEs).

Statistical analysis

All statistical analysis was performed using statistical software, SPSS version 20.0 (IBM Corp., Armonk, NY, USA). An appropriate test of significance was performed after testing the normality of the data. All the statistical tests are two-sided, and significance was determined by p<0.05.

## Results

A total of 95 patients with COVID-19 infections consented to and were screened for study eligibility. Out of these, 30 patients either failed the screening or withdrew consent during the screening process as they were not willing to continue in the trial. A total of 65 patients were enrolled in the study on day 1 after confirming eligibility; 33 patients were enrolled in the Cordyceps group and 32 in the placebo group, based on the randomization list. Out of these, seven patients (three in the Cordyceps group and four in the placebo group) did not take any medication and were excluded from the study. A total of 58 patients were thus considered evaluable for the analysis: 30 patients in the Cordyceps group and 28 in the placebo group. The mean age of all the patients enrolled in the study was 42.34 ± 13.61 years. The mean age of patients receiving Cordyceps capsules was 42.55 ± 14.71 years, and that of patients receiving Placebo was 42.12 ± 12.59 years. There was a male preponderance; 42 (64.61%) were male and 23 (35.38%) were female. The weight and height of the patients in both groups were comparable at baseline. Of the 30 patients in the Cordyceps group, 27 (90.0%) were in the mild category and 3 (10.0%) were in the moderate category. Out of 28 patients in the placebo group, 23 (82.1%) were in the mild category, and 5 (17.9%) were in the moderate category. There were no protocol deviations in the study. Six patients in the Cordyceps group had a medical history of hypertension, diabetes mellitus, and hyperthyroidism, whereas seven patients in the placebo group had a medical history of hypertension, diabetes mellitus, and ischemic heart disease. The treatment compliance of the study participants was good. Only four (17.89%) patients consumed <80% of the supplied study capsules, and 54 (83.1%) patients consumed >80% of the supplied study capsules. In seven patients, information on capsule consumption could not be retrieved.

Time to improvement of clinical symptoms

**Figure 2:**
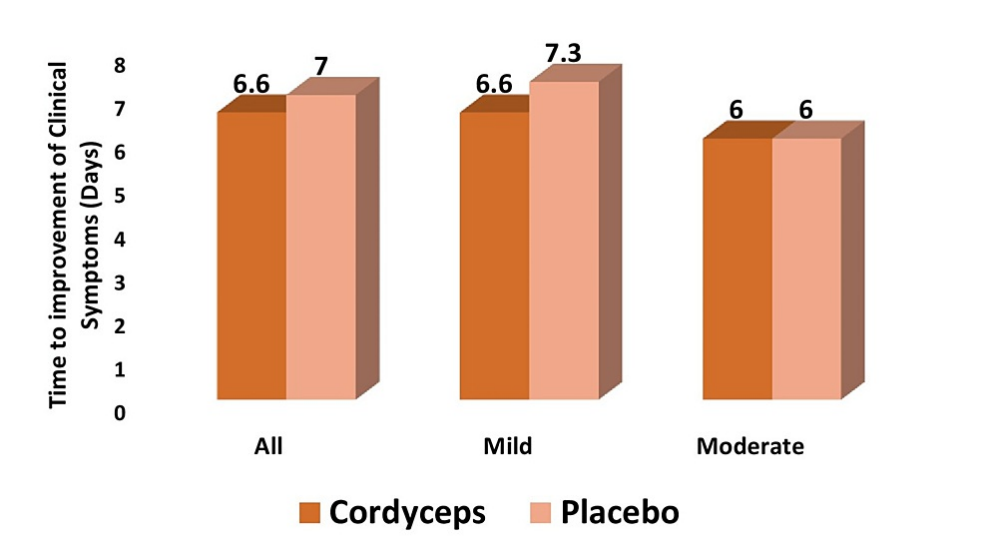
Mean time to improvement of clinical symptoms Mean time to improvement of clinical symptoms

Overall, patients receiving Cordyceps had a mean improvement in clinical symptoms earlier than patients receiving placebo (6.6 ± 2.8 days vs. 7.0 ± 3.3 days), though the difference was not statistically significant (P > 0.05) (Figure 2). In the mild category, patients receiving Cordyceps had symptoms improve a day earlier as compared to patients receiving placebo (6.6 ± 2.9 days vs. 7.3 ± 3.8 days), though the difference was not statistically significant (P > 0.05). In the moderate category, the meantime to improvement of symptoms was 6.0 ± 2.6 days in the Cordyceps group and 6.0 ± 2.1 days in the placebo group. The difference was not statistically significant (P > 0.05).

Time to recovery of clinical symptoms

The mean time to recovery of symptoms was comparable between the Cordyceps and placebo groups. Similarly, the meantime to recovery of symptoms was comparable between Cordyceps and placebo groups for mild and moderate category patients.

Improvement in individual symptoms

In both the Cordyceps and placebo groups, fever was the most common symptom on day 1 of the study, followed by cough, body pain, and weakness. Twenty-four (80%) patients in the Cordyceps group and 17 (60.7%) in the placebo group presented with fever; 17 (60.7%) patients in the Cordyceps group and 15 (53.6%) patients in the placebo group presented with cough; and 11 (36.7%) patients in the Cordyceps group and 12 (42.9%) in the placebo group presented with body pain. Weakness was present in 11 (36.7%) patients in the Cordyceps group and 8 (28.6%) patients in the placebo group. The fever subsided in all the patients (100%) on day 5 in both groups. The cough subsided in >80% of patients in both groups. Body pain subsided in all (100%) patients in the Cordyceps group and in >90% of patients in the placebo group. Weakness continues in 10% of patients in the Cordyceps group and 7.1% of patients in the placebo group on day 5, which subsequently resolves on day 10. Symptoms like breathlessness, vomiting, loss of smell and taste, headache, and sore throat were present in < 20% of patients in both groups, which resolved by day 10 in all patients.

Overall response to adjuvant treatment

Out of 58 evaluable patients, 33 (56.9%) recovered on day 5, 49 (84.5%) on day 10, and 58 (100%) on days 16 and 30. Thus, the recovery of patients steadily increased from 56.9% on day 5 to 100% on day 30.

Response to Adjuvant Treatment at Day 5

**Figure 3:**
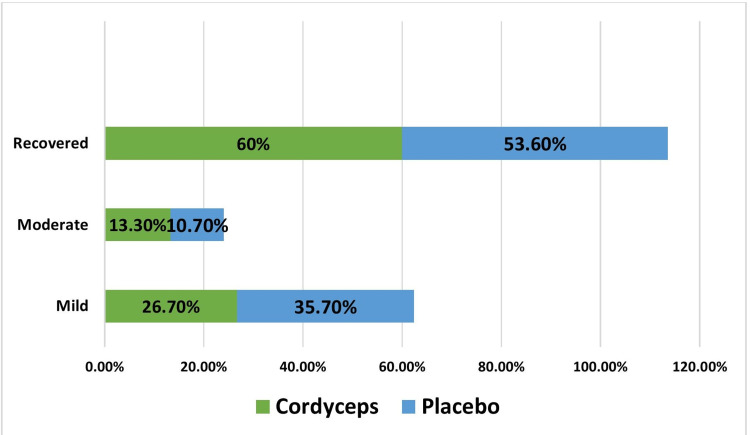
Response to treatment at day 5

A proportionately higher number of patients recovered in Cordyceps group 18 (60%) as compared to placebo group 15 (53.6%) on day 5, although the difference was not statistically significant (P>0.05) (Figure 3). Improvement was seen in mild patients. In the Cordyceps group, 18 patients (60%) recovered at day 5, while 8 (26.7%) patients remained mild and 4 (13.3%) patients remained in the moderate category (the status of two patients in the mild category changed to the moderate category). In the placebo group, 15 patients (53.6%) recovered, while 10 (35.7%) were in the mild category and 3 (10.7%) were in the moderate category.

Response to Treatment at Day 10

On day 10, similar proportions of patients recovered in Cordyceps group 25 (83.3%) and placebo group 24 (85.7%). Thus, the difference was not statistically significant (P>0.05). Improvement was seen in mild patients. In the Cordyceps group, three (10.0%) patients continued in the mild category, one (3.3%) had moderate category disease, and one (3.3%) had severe category disease. In the placebo group, 2 (7.1%) patients continued in the mild category at day 10.

Response to Treatment at Day 16

On day 16, all ongoing patients in the mild and moderate categories recovered in both groups. A proportionate number of patients recovered in Cordyceps group 30 (100%) and placebo group 28 (100%).

Response to Treatment at Day 30

On day 30, all ongoing patients in the mild and moderate categories recovered in both groups. A proportionate number of patients recovered in Cordyceps group 30 (100%) and placebo group 28 (100%).

Change in disease severity from baseline 

Change in Disease Severity at Day 5

Out of 27 mild category patients enrolled in the Cordyceps group on day 1, 18 patients improved and recovered, two patients worsened to moderate on day 3, and seven patients remained mild. Out of three patients with moderate disease, one patient improved to the mild category, whereas two patients remained moderate on day 5. Out of 23 mild category patients enrolled in the placebo group on baseline day 1, 15 patients improved and recovered, and eight patients’ disease status remained mild. Out of five patients with moderate disease, two improved to mild, whereas three patients remained moderate on day 5.

Change in Disease Severity at Day 10

In the Cordyceps group, 24 mild disease patients improved and recovered; one patient's disease status remained moderate; one patient worsened to severe; and one patient remained mild. Out of three patients with moderate disease, two improved to the mild category, and one patient recovered. In the placebo group, 21 patients improved and recovered; one patient remained mild; and one patient missed the visit. Out of five patients with moderate disease, three improved and recovered, one improved to mild, and one patient missed the visit.

Change in Disease Severity at Day 16

In the Cordyceps group, 27 patients with mild disease improved and recovered. All three patients with moderate disease improved and recovered. In the placebo group, 23 patients improved and recovered. All five patients with moderate disease improved and recovered.

Status of RT-PCR

**Figure 4:**
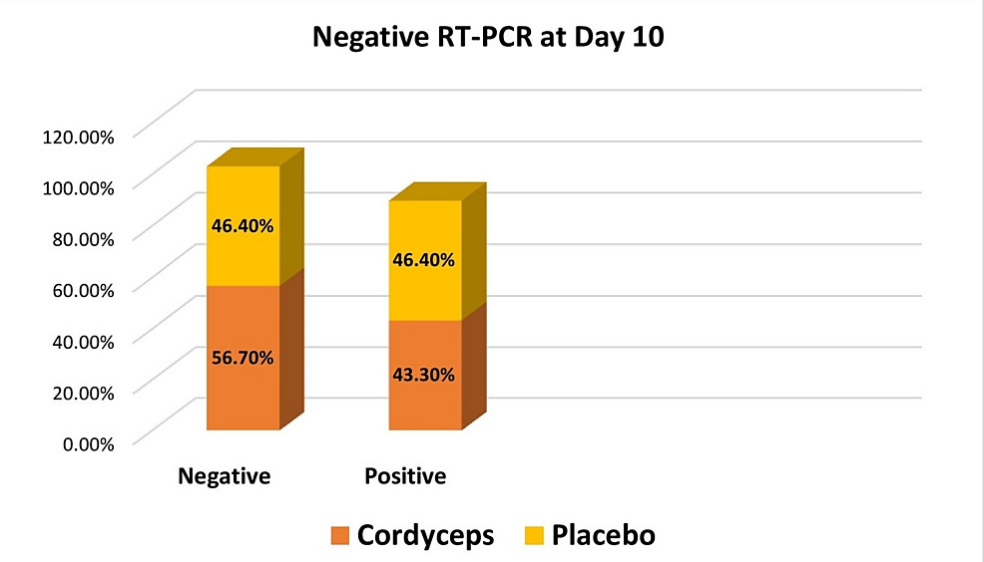
Negative RT-PCR at day 10

More than 50% of patients receiving Cordyceps showed negative RT-PCR tests on day 10. In the case of placebo, a negative RT-PCR test was seen in 46.4% of patients. Thus, a proportionately higher number of patients showed RT-PCR negative results in Cordyceps group 17 (56.7%) as compared to placebo group 13 (46.4%) on day 10, though the difference was not statistically significant (P >0.05) (Figure 4). Thirteen (43.3%) patients in the Cordyceps group and 13 (46.4%) patients in the placebo group tested RT-PCR positive on day 10. The test was not done on 2 (7.1%) patients in the placebo group as they missed their visit on day 10.

Length of hospital stay

The overall length of hospital stay for moderate-category patients was 12.5 ± 3.63 days. The mean length of hospital stay was 14 ± 3.7 days in the Cordyceps group and 11 ± 3.2 days in the placebo group. The difference in hospital stays between the two groups was not statistically significant.

Arterial blood gas analysis

Arterial blood gas analysis was done in patients with moderate-category disease. ABG parameters (pH, PaO_2_, PaCO_2_, HCO_3_, and SaO_2_) at baseline (day 1) show that pH was 7.3 in both the Cordyceps and placebo group patients. PaO_2_ was 90.5 ± 2.1 in the Cordyceps group, while it was 86.5 ± 3.5 in the placebo group. PaCO_2_, HCO_3_, and SaO_2_ were 36.0 ± 0, 22.9 ± 1.6, and 98.5 ± 0.7 in the Cordyceps group and 32.2 ± 0.7, 22.6 ± 0.3, and 95.0 ± 5.7 in the placebo group, respectively. Values of ABG parameters on day 5 were available for only two patients, and no values were available on day 10, hence the analysis was not done for these time points.

Disease biomarker assessment

Biomarkers - All Patients

There was no significant change in the mean values of IL-6, ferritin, LDH, CRP, and D-dimer levels on days 5 and 10 as compared to baseline (day 1) values in patients receiving Cordyceps capsules. Also, the comparison of IL-6, ferritin, LDH, CRP, and D-dimer between the patients receiving Cordyceps capsules and placebo capsules was not statistically significant.

There was a significant change in the mean values of MCPIP, CXCL10, and IL-1β levels on days 5 and 10 as compared to baseline (day 1) values in patients receiving Cordyceps capsules. However, similar changes were seen in the placebo group as well. There was no change in iNOS levels on days 5 and 10 as compared to baseline (day 1) values. The comparison of MCPIP, iNOS, CXCL10, and IL-1β levels between the patients receiving Cordyceps capsules and placebo capsules was not statistically significant except for CXCL10, where the comparison was significant on day 10. The values for the various biomarkers considered for all patients are shown in Table 2.

**Table 2:** Biomarkers - all patients

Biomarker	Time	Groups				P-value*
		Cordyceps		Placebo		
		N	Mean ± SD (range)	N	Mean ± SD (range)	
IL-6	Baseline (day 1)	30	24.15 ± 18.13 (3.46–94.0)	27	20.11 ± 26.36 (0.21–132.6)	0.611
	Day 5	29	19.63 ± 20.38 (3.99–70.53)	28	48.02 ± 115.06 (0.67–397.8)	0.196
	Day 10	18	24.82 ± 50.83 (2.91–226.5)	19	72.01 ± 141.99 (2.04–522.3)	0.201
	P-value/		0.404		0.13	
Ferritin	Baseline (day 1)	29	211.32 ± 239.99 (4.2–1010.5)	26	218.59 ± 340.46 (1.6–1128)	0.927
	Day 5	28	197.42 ± 240.04 (2.7–1134)	28	129.26 ± 143.06 (4.9–684.7)	0.202
	Day 10	22	153.01 ± 210.71 (2.8–907.5)	21	127.6 ± 113.07 (5.5–500.3)	0.538
	P-value/		0.338		0.445	
LDH	Baseline (day 1)	30	245.67 ± 131.5 (105–702)	27	252.00 ± 71.57 (146–440)	0.825
	Day 5	29	224.59 ± 88.51 (107–474)	27	215.59 ± 58.42 (114–363)	0.658
	Day 16	29	232.21 ± 75.06 (81–419)	24	201.63 ± 41.44 (95–311)	0.081
	P-value/		0.497		0.001	
CRP	Baseline (day 1)	30	18.48 ± 19.98 (1–69.4)	27	15.8 ± 24.91 (0.4–104.7)	0.843
	Day 5	29	15.59 ± 25.393 (0.1–105.1)	27	16.49 ± 26.76 (0.1–121)	0.898
	Day 10	24	9.16 ± 14.98 (0.12–58)	21	9.48 ± 30.48 (0.1–148.1)	0.873
	P-value/		0.135		0.751	
D-dimer	Baseline (day 1)	30	0.30 ± 0.43 (0.04–1.68)	27	0.21 ± 0.21 (0.05–0.97)	0.348
	Day 5	25	0.23 ± 0.23 (0.02–3.4)	24	0.26 ± 0.42 (0.04–2.18)	0.75
	Day 10	21	0.54 ± 1.58 (0.02–7.4)	18	0.20 ± 0.17 (0.05–0.84)	0.364
	P-value/		0.272		0.865	
MCPIP	Baseline (day 1)	30	33.1 ± 15.00 (13.28–60.08)	28	38.96 ± 19.72 (15.56–77.42)	0.207
	Day 5	29	42.98 ± 20.71 (19.92–118.20)	28	49.59 ± 24.76 (18.57–112.01)	0.278
	Day 10	30	48.79 ± 22.87 (14.92–114.76)	24	46.25 ± 18 (16.09–91.07)	0.658
	P-value/		0.001		0.125	
iNOS	Baseline (day 1)	30	1562.45 ± 755.02 (708.14–4587.45)	28	1356.61 ± 556.85 (621.12–3034.81)	0.245
	Day 5	29	1726.58 ± 498.36 (715.31–3003.63)	28	2102.96 ± 1735.09 (684.85–8196.56)	0.267
	Day 10	30	2022.81 ± 1586.56 (766.59–9039.4)	24	2174.54 ± 2080.95 (789.61–11424.12)	0.762
	P-value/		0.206		0.207	
CXCL10	Baseline (day 1)	30	347.03 ± 127.76 (138.99–789.57)	28	294.79 ± 116.86 (96.78–498.68)	0.111
	Day 5	29	263.15 ± 102.85 (115.55–102.85)	28	216.6 ± 101.44 (64.58–450.77)	0.091
	Day 10	30	202.19 ± 88.73 (87.90–478.12)	24	156.51 ± 71.03 (46.90–789.0)	0.045
	P-value/		<0.0001		<0.0001	
IL-1β	Baseline (day 1)	30	84.73 ± 81.06 (18.52–459.60)	28	59.29 ± 31.62 (17.53–150.36)	0.126
	Day5	29	46.38 ± 33.28 (18.81–178.62)	28	39.42 ± 21.21 (17.95–112.67)	0.352
	Day10	30	36.3 ± 28.34 (12.57–115.63)	24	44.42 ± 26.79 (12.68–97.62)	0.289
	P-value/		0.005		0.042	

Biomarkers - Moderate Category Patients

There was a significant change in the mean value of CRP levels on day 5 as compared to baseline (day 1) values in patients receiving Cordyceps capsules. Also, there was a decrease in IL-6 values on days 5 and 10, but because of the very small number of patients (n=2), statistical comparison was not possible. There was no significant change in ferritin, LDH, or d-dimer levels on days 5 and 10 compared to baseline values. The comparison of IL-6, ferritin, LDH, CRP, and D-dimer between the moderate category patients receiving Cordyceps capsules and placebo capsules was not statistically significant. There was a significant change in the mean values of CXCL10 on day 10 as compared to baseline (day 1) values in moderate category patients receiving Cordyceps capsules. However, this change was seen in the placebo group as well. There was no change in the MCPIP, iNOS, and IL-1β levels in moderate category patients on days 5 and 10 as compared to day 1 values. The values for the various biomarkers considered for moderate-category patients are shown in Table 3.

**Table 3:** Biomarkers - moderate category patients

Biomarker	Time	Groups				P-value*
		Cordyceps		Placebo		
		N	Mean ± SD (range)	N	Mean ± SD (range)	
IL-6	Baseline (day 1)	3	29.35 ± 4.06 (25.0–33.0)	5	21.59 ± 9.31 (22.8–33.3)	0.229
	Day 5	3	13.13 ± 8.86 (5.1–22.6)	5	116.52 ± 215.78 (7.0–31.1)	0.452
	Day 10	2	17.09 ± 5.5 (13.2–20.9)	2	29.35 ± 6.48 (3.48–33.9)	0.178
Ferritin	Baseline (day 1)	3	358.67 ± 185.52 (182.5–552.3)	5	428.72 ± 490.28 (73.2–1128)	0.825
	Day 5	3	610.8 ± 454.93 (308–1134)	5	198.24 ± 274.8 (44–684.7)	0.153
	Day 10	2	325.9 ± 290.9 (120.2–531)	2	275.65 ± 317.7 (20.6–500.3)	0.884
LDH	Baseline (day 1)	3	336 ± 199.59 (188–563)	5	318.8 ± 85.18 (220–415)	0.867
	Day 5	3	285 ± 169.37 (132–467)	5	257.8 ± 59.62 (220–363)	0.745
	Day 16	3	297.33 ± 146.3 (135–419)	4	215.75 ± 68.42 (165–311)	0.362
	P–value/		0.566		0.106	
CRP	Baseline (day 1)	3	40.07 ± 10.67 (28.6–49.7)	5	34.62 ± 30.16 (10.1–86)	0.779
	Day 5	3	6.53 ± 1.7(4.9–8.3)	4	48.18 ± 48.64 (12.9–121)	0.208
	Day 10	3	20.13 ± 19.05 (1.3–39.4)	3	51.83 ± 83.37 (2–148.1)	0.556
	P-value/		0.049		0.687	
D-Dimer	Baseline (day 1)	3	0.55 ± 0.45 (0.1–0.99)	5	0.23 ± 0.18 (0.06–0.52)	0.187
	Day 5	3	0.63 ± 0.47 (0.09–0.9)	3	0.23 ± 0.15 (0.04–0.36)	0.235
	Day 10	3	0.2 ± 0.15 (0.06–0.36)	3	0.39 ± 0.39 (0.1–0.8)	0.466
	P-value		0.178		0.415	
MCPIP	Baseline (day 1)	3	51.11 ± 11.09 (38.35–58.41)	5	36.61 ± 24.63 (15.56–77.42)	0.383
	Day 5	3	51.31 ± 14.93 (35.91–65.73)	5	48.67 ± 12.38 (34.59–63.48)	0.795
	Day 10	3	65.4 ± 29.08 (32.30–86.87)	3	35.96 ± 18.16 (19.81–55.61)	0.211
	P-value		0.701		0.914	
iNOS	Baseline (day 1)	3	2676.71 ± 1849.77 (894.62–4587.45)	5	1458.99 ± 469.97 (843.71–1943.34)	0.192
	Day 5	3	1759.58 ± 328.04 (1381.36–1966.63)	5	2229.86 ± 1067.31 (1550.15–4104.67)	0.497
	Day 10	3	1859.38 ± 795.9 (1142.16–2715.64)	3	1897 ± 142.14 (1762.41–4640.71)	0.94
	P-value		0.408		0.506	
CXCL10	Baseline (day 1)	3	494.12 ± 76.43 (406.44–546.68)	5	366.81 ± 148.86 (168.52–498.68)	0.226
	Day 5	3	351.57 ± 76.44 (298.51–439.18)	5	289.93 ± 149.3 (118.68–450.77)	0.539
	Day 10	3	204.9 ± 51.34 (145.62–234.68)	3	234.46 ± 102.07 (125.68–345.68)	0.677
	P-value		0.010		0.009	
IL-1β	Baseline (day 1)	3	205.71 ± 219.87 (78.64–459.60)	5	52.19 ± 30.47 (19.67–87.56)	0.155
	Day 5	3	44.02 ± 18.46 (22.84–56.65)	5	31.63 ± 10.12 (20.96–44.09)	0.255
	Day 10	3	26.68 ± 12.23 (12.57–34.23)	3	36.8 ± 17.28 (25.36–56.68)	0.454
	P-value		0.260		0.857	

IgG analysis at day 16

The mean IgG levels at day 16 were 26.86 ± 27.45 in the Cordyceps group and 16.52 ± 22.86 in the placebo group, with a p-value of 0.141 obtained using a t-test for independent samples. Although this is a huge difference numerically, it did not reach the significance level since the numbers of patients were lower for such a study.

Adverse events

Overall, 10 (17.2%) patients developed 16 treatment-emergent adverse events (TEAEs). Out of these, 12 TEAEs were reported in 6 (20.0%) patients receiving Cordyceps capsules, and 4 TEAEs were reported in 4 (14.3%) patients receiving placebo capsules. Nine TEAEs were treatment-related. Of these, 5 TEAEs were related to Cordyceps capsules, and 4 TEAEs were related to placebo capsules. Three (10.0%) patients in the Cordyceps group and four (14.3%) patients in the placebo group reported the related TEAEs. None of the patients in the study developed severe or serious TEAEs. Two patients had drug interruptions due to the progression of their disease into the moderate category. All TEAEs were resolved with or without medication. None of the subjects developed any serious adverse events in this study.

Clinical examination, laboratory parameters, and investigations

The haematology parameters in the Cordyceps group did not change significantly on days 5 and 16 from the baseline (day 1) visit except for WBC count, platelet count, and eosinophil count. These counts change significantly on day 16 as compared to day 1 values but do not have any clinical significance. There was no significant difference in the haematology parameters between the patients receiving Cordyceps capsules and placebo capsules at baseline (day 1), day 5, and day 16, except for neutrophil count and lymphocyte count. Overall, it is observed that there is no impact of the drug on haematology parameters. The biochemistry parameters in the Cordyceps group did not change significantly on days 5 and 16 from the baseline (day 1) visit except ALP, ALB, and BUN. Although these values changed significantly on days 5 and 16 as compared to day 1 values, they do not have any clinical significance. There was no significant difference in the biochemistry parameters between the patients receiving Cordyceps capsules and placebo capsules at baseline (day 1), day 5, and day 16. Overall, it is observed that the drug has no impact on biochemical parameters. The urine parameters did not change significantly on days 5 and 16 from the baseline visit in patients receiving Cordyceps capsules. Similarly, there was no significant difference in the urine parameters between the patients receiving Cordyceps capsules and placebo capsules, indicating there is no significant impact of the drug on these parameters.

Overall, physical examination of patients was normal at all visits

On baseline (day 1), 53.3% of patients in the Cordyceps group and 64.3% in the placebo group had normal chest X-rays (Table 4). Other important findings were bilateral lower-zone infiltrates and ground-glass opacities in the bilateral lower zones. On day 16, all patients had normal chest X-rays. ECG findings were normal in most of the patients at baseline (day 1) and day 16 visits.

**Table 4:** Distribution of patients as per X-ray findings in two groups at two time points

Finding	Groups	
	Cordyceps	Placebo
Baseline (day 1)	N=30	N=28
Normal	16 (53.3%)	18 (64.3%)
Bilateral lower zone infiltrates	3 (10.0%)	3 (10.7%)
Ground glass opacities bilateral lower zones	2 (6.7 %)	0 (0)
Ground glass opacities bilateral mid and lower zone	3 (10.0%))	3 (10.7%))
Hyperinflation	1 (3.3%)	0 (0)
Linear reticular opacities bilateral para hilar paracardial region	1 (3.3%)	0 (0)
Lower zone infiltrates	0 (0)	1 (3.6%)
Mild ground glass opacities bilateral lower zones	1 (3.3%)	1 (3.6%)
Mild ground glass opacities bilateral mid and lower zones	1 3.3%)	1 (3.6%)
Prominent pulmonary vessels	1 (3.3%)	1 (3.6%)
Unfolding of aorta	1 (3.3%)	0 (0)
Day 16		
Normal	30 (100%)	25 (89.3%)*

## Discussion

There are two species of Cordyceps that are used for treatment: C. sinensis and C. militaris. Cordyceps affects the immune system [22-24]. It increases the responsiveness and intracellular signalling in macrophages and antigen-presenting cells by getting spotted by toll-like receptors and c-type lectin receptors, natural killer cells, stimulates T-cells, increases the numbers of CD4+ and CD8+ cells, and increases the production of IFN-γ [23]. In the context of SARS-CoV2, C. militaris and C. sinesis containing unique nucleosides have been studied for their anti-viral potential. In addition to nucleosides, these medicinal funguses also contain some important phytoconstituents that can be beneficial in the prevention and treatment of the infection and the management of symptoms associated with COVID-19 patients [2-8]. Cordycepin (3'-dA) is a purine nucleoside lacking a 3'-hydroxyl group of adenosines. Its antiviral property has been extensively studied by various research groups against the pi-coronavirus family [12], HIV [25], Epstein-Barr virus [26], and influenza [27,28] in the last few decades. The anti-inflammatory potential of Cordycepin has been evident in many published reports. Cordycepin downregulates iNOS, COX-2, and TNF-α gene expression via the suppression of NF-κB activation and phosphorylation of protein kinases, i.e., Akt and p38 [10]. Literature evidence indicates that the anti-viral activity of Cordyceps, i.e., Picorna viruses (RNA viruses), is due to inhibition of RNA synthesis [12,13]. Cordyceps has been shown to protect the lungs from various pathological conditions, mainly through its anti-inflammatory effects [17,22,29]. In a study, patients with moderate to severe asthma performed better and had improved lung function and quality of life while taking Cordyceps supplements. The treatment group had a significant increase in AQLQ scores and lung function in comparison to the control group. The expression levels of the inflammation markers in the serum for IgE, ICAM-1, IL-4, and MMP-9 were decreased, and IgG was increased in the treatment group in comparison to the control group [17,22]. Another study showed that asthma patients who were on Cordyceps supplements had lower levels of lab markers for airway inflammation compared to those who were not on the supplement [11]. Cordyceps has also been shown to improve lung function in COPD patients [10,27,28]. In a rat model, cordyceps showed reduced airway thickening, inflammatory cell buildup, and cytokine production [19]. Animal studies also suggested that Cordyceps may help protect the lungs from cigarette smoking damage [19,20].

Animal studies have shown that Cordyceps extract and its isolated active components may help fight flu viruses. Cordyceps has been found to increase immune system activity and improve survival rates in animal studies [27,30]. Thus, the same agent may have similar health benefits for humans affected by COVID-19. In our study, we found that treatment with adjunct Cordyceps over standard treatment alone improved recovery rates at days 5 and 16, with less time for clinical and radiological recovery. The hastened recovery seen with adjunct Cordyceps was seen for overall cases and mild infections; however, for moderate infections, it was similar to the standard treatment. Cordyceps contains a wide range of nutrients, including various types of essential amino acids, vitamins like Bl, B2, B12, and K, different kinds of carbohydrates, and proteins, which therefore assist in better recovery from the infection [31]. Clinical studies suggest that Cordyceps shows clinical effectiveness in alleviating fatigue and improving physical endurance, especially in elderly subjects, by increasing the cellular bio-energy ATP and improving the efficient utilisation of oxygen [32]. Therefore, it may help maintain the stamina of patients by sustaining normal cellular metabolic processes through the effective utilisation of oxygen.

Influenza viruses are primarily responsible for seasonal flu cases occurring during flu season. Evolutionarily, the influenza viruses are distinct from the beta coronaviruses, and infections with the influenza virus do not provide cross-immunity with the beta coronaviruses. In this context, consistent with studies depicting the co-infection with influenza A and SARS-CoV-2 viruses, Leyfman et al. [33] proposed the conceptual framework of "COVI-FLU" and suggested that synergistic infection results in widespread organ damage due to cytokine release syndrome. Both viruses have different attachments to the cell surface and cause pulmonary damage and IL-6 release; however, the SARS-CoV-2 virus can result in systemic damage in addition due to the presence of ACE2 receptors in other organ systems as well. Through indirect mechanisms, the virus can recruit neutrophils and macrophages in the pulmonary and other organ systems, resulting in systemic damage. This has formed the basis for several single-agent therapeutic drugs targeting the direct and indirect damage pathways in SARS-CoV-2. As the level of inflammatory markers decreased in the Cordyceps group in our study, it might be possible that the anti-inflammatory properties of Cordyceps may help reduce the systemic damage occurring due to the indirect pathway of cytokine release syndrome.

On the other hand, the word "cytokine storm" has triggered a debate in the medical literature. This is mainly because cytokine storm does not truly reflect the severity of the disease or the likelihood of improvement with cytokine inhibitors [34]. Stolarski et al. [35] published in their study that the concentration of IL-6 was much lower in severe SARS-CoV-2 disease than in septic shock. The authors further reported that, though different cytokine levels were much higher in septic shock, patients have not improved with anti-cytokine therapies. On the other hand, the levels of cytokines were much lower in autoimmune diseases, and patients responded well to anti-cytokine management [35]. Therefore, the exact mechanisms of how the therapeutic agents protect against systemic damage in SARS-CoV-2 are poorly understood and need more exploratory immunological studies.

Limitations

Although we have predefined exclusion criteria to avoid most of the confounding factors, the possibility of interaction with a confounder that cannot be easily controlled in the analysis cannot be ruled out. The radiological densities could be secondary to previous pulmonary infections, including tuberculosis and cardiac involvement in COVID infection, and thus may influence the results we have obtained. The group sizes are small, and a larger group size might show the significance that was not present in the early trends in this report. In the present report, there are trends for better clinical improvement in moderate to severe COVID infection with the use of *C. militaris*, but significance was not achieved. This could be due to the small size of the individual groups and also unknown factors that might influence the results, as COVID has been a relatively recent infection and many facts are yet to be deciphered. Though the results were non-significant, these were early trends that need to be further studied by studies with larger sample sizes and different study populations.

## Conclusions

Cordyceps capsules administered at a dose of 500 mg three times a day along with supportive treatment showed early trends in effectiveness in patients with mild to moderate COVID-19 infection, as evidenced by the proportionately higher number of recoveries on day 5, the relatively shorter time for improvement of clinical symptoms, and the proportionately higher number of patients showing negative RT-PCR tests on day 10. Significant changes were seen in biomarkers MCPIP, CxCL10, and IL-1β for overall (both mild and moderate patients) on days 5 and 10 as compared to baseline, and in biomarkers CRP and CxCL10 for moderate category patients on days 5 and 10, respectively. Recovery of symptoms was mainly seen in mild patients, wherein patients receiving Cordyceps had symptoms improve earlier as compared to patients receiving a placebo. No significant worsening of the disease-related markers such as CRP, IL-6, ferritin, and D-dimers was observed. However, some biomarkers of inflammation, for example, ferritin, LDH, CXCL-10, and D-dimer, increased on day 5 in the Cordyceps group, but none of the increases reached statistical significance. The medication was safe and well tolerated by patients, with no drug interruption or dose reduction due to minor adverse events in any of the patients. Though major endpoints in the study did not reach clinical significance, the changes in the groups were substantial. But the early trends observed suggest the potential benefit of *C. militaris* and need to be confirmed in other studies with larger groups. Overall, the study results suggested that Cordyceps can be a safe immunological adjuvant in the treatment of patients with mild to moderate COVID-19 infection.
